# Neonatal jaundice detection using a vision transformer-based deep learning model

**DOI:** 10.1038/s41598-026-40515-5

**Published:** 2026-02-16

**Authors:** Mehrnoush Lotfi, Mohammad Rabiee, Masoomeh Haghbin Nazarpak, Razieh Sangesari, Nazanin Alishahi, Mohammad Saber Azimi

**Affiliations:** 1 Biomaterials Group, Department of Biomedical Engineering, Amirkabir University of Technology, Tehran, Iran; 2 Division of Neonatology, Department of Pediatrics, Pediatric Center of Excellence, Faculty of Medicine, Children’s Medical Center, Tehran University of Medical Sciences, Tehran, Iran; 3 Department of Medical Radiation Engineering, Shahid Beheshti University, Tehran, Iran

**Keywords:** Neonatal jaundice, Vision transformer (T2T-ViT), Deep learning, Skin imaging, Non-invasive bilirubin estimation, Neonatology, Paediatric research, Biomedical engineering, Skin diseases, Health care

## Abstract

Neonatal jaundice is a prevalent and potentially serious condition that can lead to severe complications if undiagnosed or untreated. While traditional diagnostic methods like blood sampling are invasive and time-consuming, and transcutaneous bilirubinometers remain costly, smartphone-based image analysis offers a promising low-cost, non-invasive alternative. However, most existing solutions rely on traditional machine learning techniques with limited accuracy and generalizability. In this study, we introduce a deep learning approach based on the Vision Transformer (T2T-ViT) and compare its performance with three other models, ResNet, Support Vector Machine (SVM), and K-Nearest Neighbors (k-NN), using a clinically annotated dataset of neonatal skin images captured via a smartphone camera. The models were evaluated using multiple performance metrics including accuracy, precision, recall, F1-score, Matthews Correlation Coefficient (MCC), and Area under the Curve (AUC). The T2T-ViT model achieved 99% across all metrics, significantly outperforming both convolutional and traditional machine learning models. These findings demonstrate the feasibility of applying transformer-based deep learning architectures for accessible, scalable, and accurate non-invasive neonatal jaundice screening, potentially enabling early intervention in resource-limited settings. This approach could serve as an accessible, scalable screening tool for neonatal jaundice detection, particularly in low-resource clinical settings.

## Introduction

Neonatal jaundice, described in medical literature for centuries, with an early reference in Bartholomaeus Metlinger’s Ein Regiment der Jungen Kinder (1473), affects 60% of term and up to 80% of preterm infants^1,2^. It manifests as yellow discoloration of the skin and sclera due to elevated serum bilirubin. Although often transient, unrecognized or untreated cases can progress to severe hyperbilirubinemia with acute bilirubin encephalopathy and kernicterus; a decade ago, hyperbilirubinemia was estimated to contribute to > 114,000 neonatal deaths and 75,000 cases of long-term neurologic disability worldwide^3,4^. These figures underscore the need for timely detection and monitoring, typically managed with phototherapy and, in severe cases, exchange transfusion^5^. Yet visual assessment is subjective and laboratory TSB measurement is invasive and resource-intensive, motivating accessible, non-invasive screening approaches that can complement guideline-based care^6^.

Several non-invasive alternatives to serum testing have been developed, including transcutaneous bilirubin meters such as BiliCheck and JM-102 Minolta^7–10^, as well as smartphone-based applications like BiliCam, BiliScan, Biliscreen, and BiliCapture^11–15^. While these approaches reduce reliance on invasive sampling, their adoption remains limited due to cost, accessibility, and sensitivity to variations in lighting and skin tone^16,17^. To improve reliability, researchers have explored a variety of anatomical sites for image-based jaundice detection, including the face^18^, forehead^11,19–21^, sternum^11–13,20^, abdomen^13,22^. Some studies have combined multiple regions such as the palm, sole, arm, and forehead^23,24^, while others have targeted the sclera as a sensitive marker of bilirubin levels^14,15,25–28^. A few investigations have even examined stool color as an indirect indicator^29^. Although these approaches demonstrate creativity and clinical relevance, their performance has often been inconsistent, largely due to lighting conditions, skin pigmentation, and device variability. While artificial intelligence (AI) and image analysis have shown promise for non-invasive jaundice detection, most prior studies relied on conventional CNNs, which primarily capture local features but struggle with long-range dependencies and subtle global patterns^30–34^. These limitations are particularly problematic in neonatal jaundice, where mild cases often present with subtle chromatic changes that can be missed by local-only feature extraction. This research gap highlights the need for architectures capable of modeling both local and global context.

Several recent studies have explored deep learning and smartphone-based methods for neonatal jaundice detection. Nayagi and Angel^35^applied convolutional neural networks (CNNs) with color card techniques to improve diagnostic accuracy. Makhloughi proposed a 1D CNN for non-invasive bilirubin prediction, demonstrating the feasibility of lightweight neural architectures^36^. Park et al. developed a smartphone-based application for scleral jaundice detection in hepatobiliary and pancreatic diseases, highlighting the potential of mobile imaging for early diagnosis^37^. Similarly, Althnian et al. employed transfer learning on fused eye and skin features captured by smartphones for neonatal jaundice screening^38^. While these studies highlight the promise of AI for jaundice detection, they remain largely CNN-based and thus limited in capturing broader contextual dependencies. Building on these insights, our work explores a Vision Transformer architecture as an alternative approach.

**Fig. 1:**
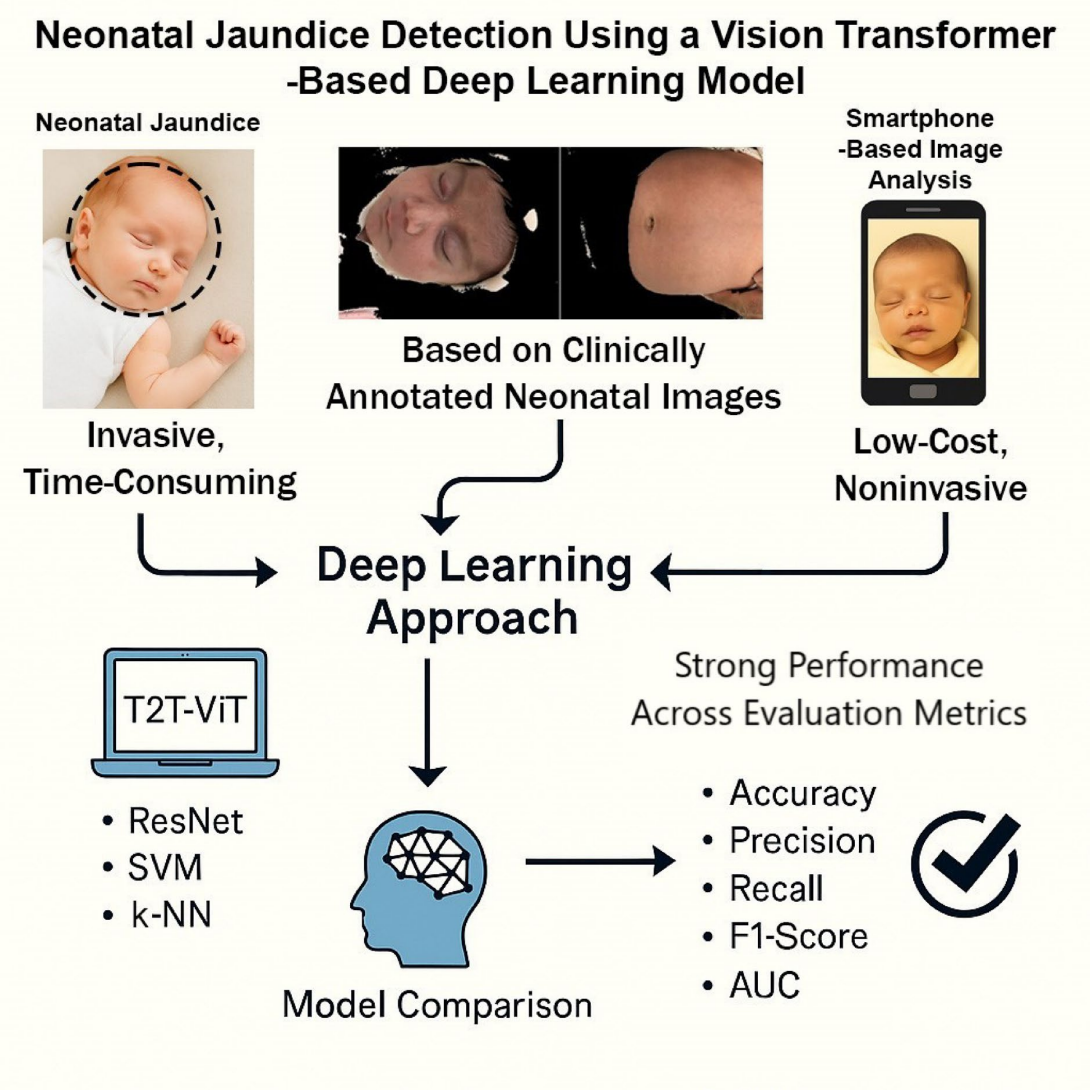
Overview of the proposed smartphone-based framework for neonatal jaundice detection. Clinically annotated neonatal skin images acquired using a smartphone camera are processed through a preprocessing pipeline and analyzed using a Tokens-to-Token Vision Transformer (T2T-ViT). The proposed approach is benchmarked against conventional deep learning and classical machine learning models for classification performance evaluation.

Although CNN-based methods and transfer learning approaches have demonstrated feasibility, they remain constrained by reliance on local feature extraction, limited validation across populations and devices, and evaluation under tightly controlled conditions. These limitations are particularly critical in neonatal jaundice detection, where subtle chromatic differences must be captured to identify mild cases. Transformer-based architectures such as the Tokens-to-Token Vision Transformer (T2T-ViT) address these challenges by leveraging attention mechanisms to integrate both local and global dependencies, improving robustness to lighting variation, background artifacts, and skin tone diversity. To address these challenges, we propose a deep learning framework for neonatal jaundice detection using skin images captured via smartphone. The framework centers on the T2T-ViT, a lightweight yet powerful transformer architecture, and is benchmarked against a conventional CNN (ResNet-50) and classical machine learning models including Support Vector Machine (SVM) and k-Nearest Neighbors (k-NN). All models were trained and evaluated on a clinically curated dataset of neonatal skin images collected under standardized imaging conditions at the Children’s Medical Center in Tehran, Iran. An overview of the proposed framework is illustrated in Fig. 1. The main contributions of this paper are summarized as follows:

- Application of Vision Transformers – Introduction of T2T-ViT for neonatal jaundice detection, among the first transformer-based applications in this field.
- Smartphone-based pipeline – Development of a simple, non-invasive, and smartphone-compatible framework validated on real neonatal data.
- Comparative evaluation – Head-to-head benchmarking against CNN (ResNet-50) and classical ML models (SVM, k-NN), showing the superior performance of attention-based models.
- Clinical context and future directions – Discussion of clinical implications, limitations, and strategies for improving generalizability across diverse devices and populations.

## Materials and methods

### Ethical approval and participant recruitment

The study followed the principles of the Declaration of Helsinki^39^and received approval from the Institutional Review Board of the Children’s Medical Center, Tehran University of Medical Sciences (project code: IR.TUMS.CHMC.REC.1399.001). Written informed consent was obtained from the parents or legal guardians of all neonates prior to enrollment. To ensure voluntary participation, the study procedures, including image acquisition and blood sampling, were clearly explained to families in advance.

Eligible participants were term or near-term neonates with a gestational age of 35–42 weeks, postnatal age between 1 and 5 days, and a birth weight of 2.0–4.35 kg. Only infants who were calm and not irritable during imaging were included, in order to reduce motion artifacts and maintain consistency across images. Exclusion criteria were ongoing phototherapy, admission to the neonatal intensive care unit (NICU), or the presence of major congenital anomalies, infections, or unstable clinical conditions. Each infant was imaged only once to avoid variability due to treatment effects or natural bilirubin decline. For all enrolled neonates, demographic and clinical data were documented, and personal identifiers were removed to maintain confidentiality.

### Data collection and image acquisition

Between July 2023 and February 2024, 500 neonates were recruited from the Laboratory Department of the Children’s Medical Center in Tehran, Iran. Of these, 193 were clinically diagnosed with jaundice and 307 served as healthy controls (Table 1). Diagnosis was based on total serum bilirubin (TSB) levels interpreted using standard pediatric nomograms. Each neonate was assigned a unique anonymized code, and both clinical and imaging data were stored securely with encrypted access.

Image acquisition was performed using the rear-facing 12-megapixel camera of an Apple iPhone 6 S mounted on a fixed tripod at a distance of 30 cm. The native iOS camera application was used with default settings, and neither flash nor external optical accessories (e.g., lenses or ring lights) were applied. All photographs were obtained in a dedicated imaging room illuminated by two 46-watt compact fluorescent lamps, providing consistent and uniform lighting. The room was windowless, preventing external light interference, and the lamps were routinely checked to maintain stability over the collection period.

**Fig. 2:**
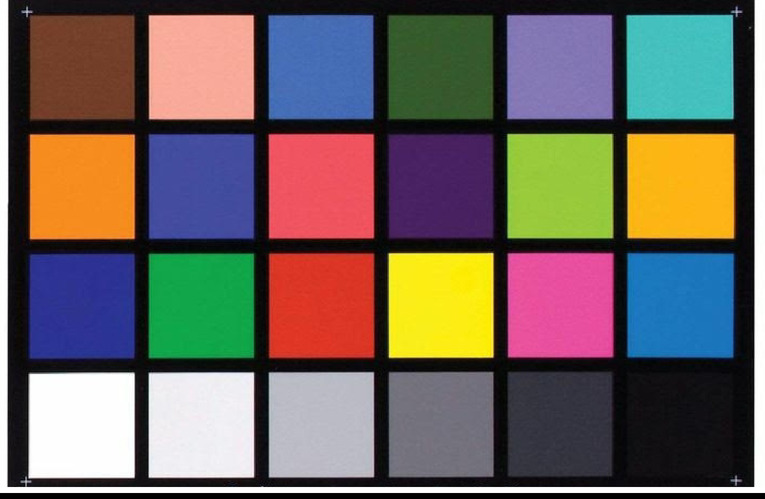
Color calibration card, placed adjacent to the neonate’s skin, and used to standardize color representation and ensure consistency across all samples.

For each neonate, three anatomical regions were imaged: the face, abdomen, and inner forearm. A 24-color calibration card with grayscale patches was placed adjacent to the skin in every frame to provide a reference for chromatic normalization. Although not from a commercial brand, the same card was consistently used across the dataset to reduce inter-sample variation caused by lighting or device settings and to support color correction during preprocessing (Fig. 2). Four photographs were taken per subject, and the clearest image was selected for analysis. All images were captured in RAW format without compression to preserve color fidelity. A trained research assistant reviewed each image, excluding those with poor focus, motion blur, or improper framing. If no adequate replacement was available, the case was omitted from the dataset.

### Ground truth labeling and blood sampling

To establish the ground truth for bilirubin levels, blood sampling was performed for every neonate within five minutes of image acquisition. Samples were collected by trained clinical staff from the forearm using standard scalp vein sets and were immediately analyzed in the hospital’s central laboratory. Bilirubin levels were measured with a Hitachi 917 Biochemistry Analyzer using kits from Kitman Diagnostics. Each measured TSB value was securely recorded and linked to the corresponding image ID.

**Fig. 3:**
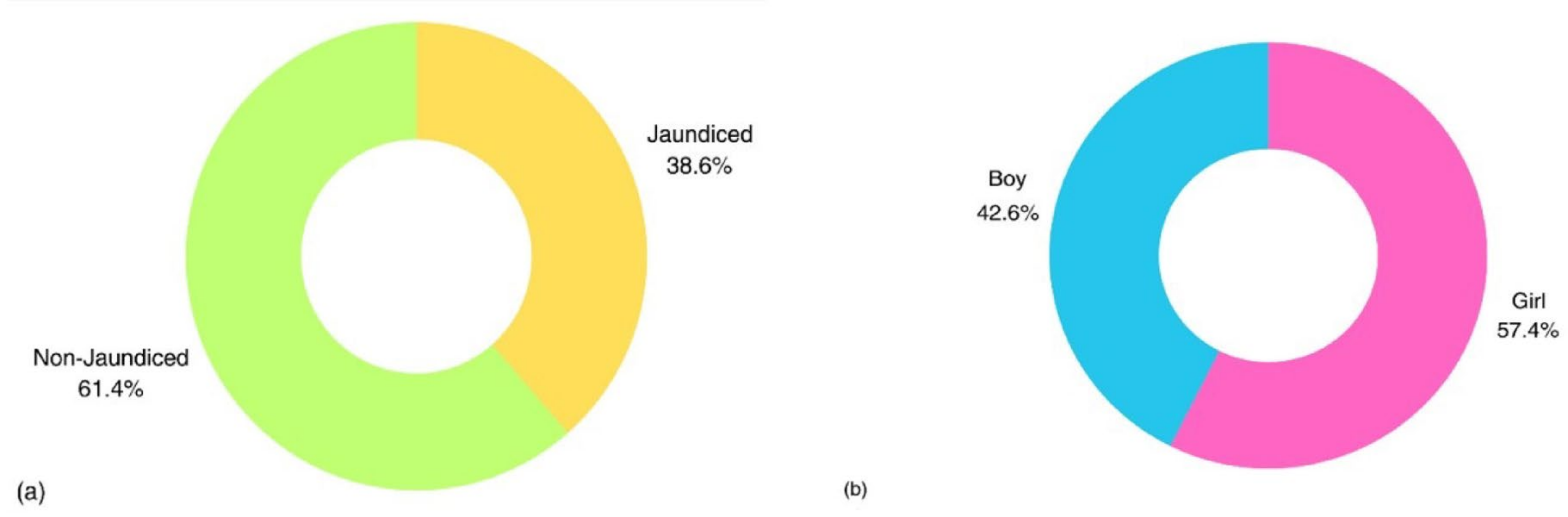
Distribution of the neonatal population by jaundice statue (**a**), and gender (**b**), showing the overall balance between male and female infants and the proportion of jaundiced cases within each group.

Jaundice classification was performed according to clinical thresholds routinely used in neonatal care in Iran determined by neonatologists based on a combination of TSB levels, postnatal age, and gestational age, consistent with internationally recognized guidelines. A bilirubin threshold of 15 mg/dL was used to define jaundice status, in line with clinical guidelines for term neonates aged 1–5 days. As illustrated in Fig. 3, the dataset included both male and female neonates with varying jaundice status, ensuring a balanced and representative population. Infants with serum bilirubin levels equal to or above this threshold were classified as jaundiced, while those below were considered non-jaundiced. This cutoff is commonly used in clinical practice to guide further diagnostic evaluation and potential phototherapy While the raw TSB values were preserved for potential regression modeling in future work, this study focused on binary classification to assess the feasibility of automated jaundice screening. Diagnostic labels were verified by a neonatologist unaffiliated with the data collection team to minimize bias. Infants were labeled as jaundiced if their TSB, measured by standard laboratory assay on the same day as imaging, was ≥ 15 mg/dL; otherwise, they were labeled non-jaundiced. The dataset comprised 792 images in total, which were divided into training (634) and test (158) subsets using an 80/20 split. To mitigate class imbalance, the training set was balanced through targeted augmentation, yielding 317 jaundiced and 317 non-jaundiced images. The test set preserved natural prevalence without augmentation (61 jaundiced, 97 non-jaundiced) and included no augmented samples, thereby ensuring unbiased evaluation and reflecting realistic screening conditions.

**Table 1:** Clinical characteristics of neonatal dataset used for model development, including number of babies, age and distribution of jaundiced/non-jaundiced cases.

Characteristics	Value
Number of babies	500
Positive Jaundice	193
Negative Jaundice	307
Male	287
Female	213
Max. age (days)	5
Average age (days)	3
Min. age	1

### Image preprocessing

**Fig. 4:**
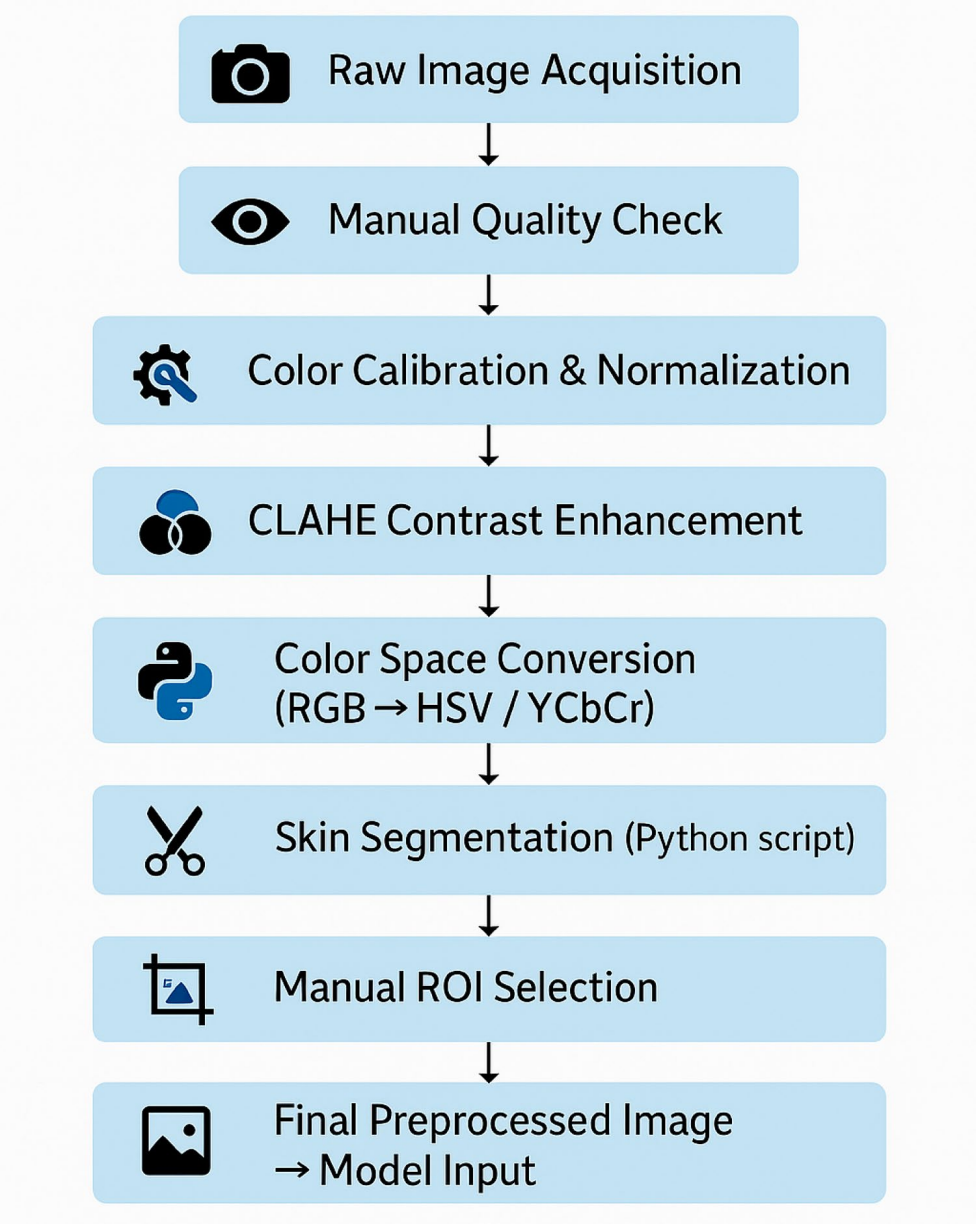
Overview of the image preprocessing pipeline for neonatal skin images. The process includes raw image acquisition, manual quality control, color calibration, contrast enhancement using CLAHE, conversion to HSV and YCbCr color spaces, skin segmentation via Python scripting, and manual selection of regions of interest (ROIs). The resulting preprocessed images are standardized and resized before being used as input for deep learning models.

Preprocessing of neonatal skin images is a critical step for achieving accurate and reproducible results in jaundice detection. In this study, we implemented a structured preprocessing pipeline aimed at improving image quality, minimizing background noise, and isolating the regions of interest (ROIs) required for analysis. The pipeline consisted of sequential steps, including quality control, color normalization, contrast enhancement, conversion to appropriate color spaces, skin segmentation, and ROI extraction. An overview of the preprocessing workflow is presented in Fig. 4.

#### Image quality control and cleaning

**Fig. 5:**
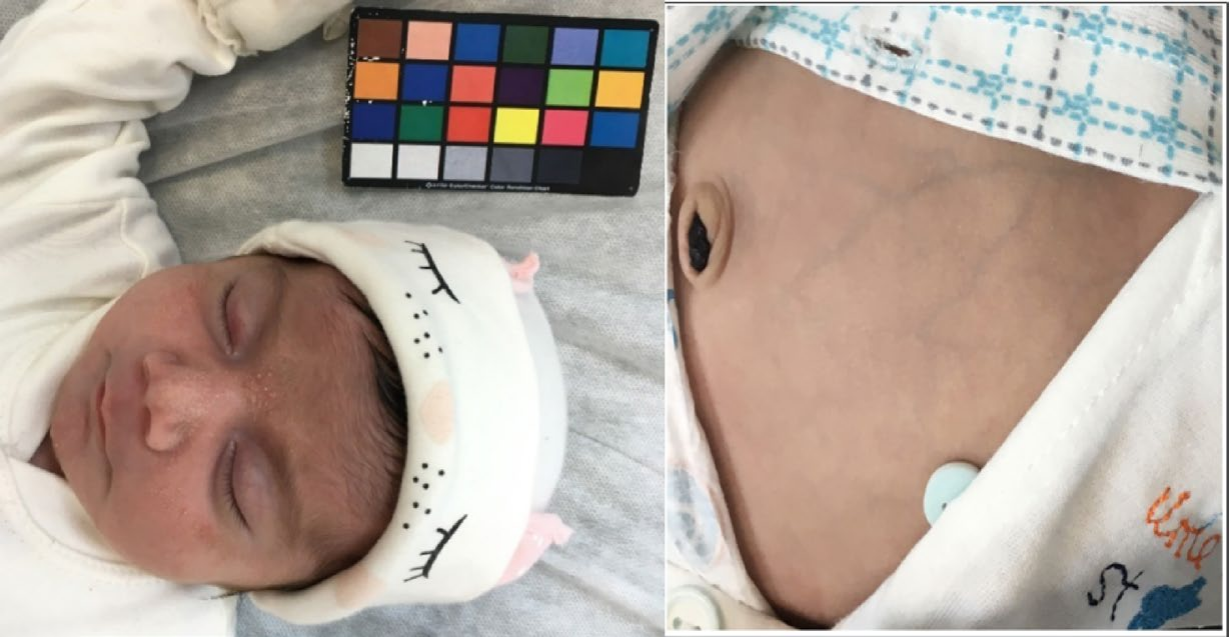
Initial input image for Preprocessing. This image includes the complete details of the infant’s face and body, captured before applying any processing for diagnosis and image enhancement.

After acquisition, each image was manually inspected by trained staff to ensure adequate quality. Photographs affected by motion blur, poor illumination, incorrect framing, or insufficient contrast were excluded. To maintain color fidelity and allow uniform preprocessing, the remaining images were stored in RAW format. Figure 5 illustrates an example of an unprocessed image, showing the infant’s face and body prior to any preprocessing steps.

#### Color calibration and normalization

A color calibration card was included in each image to allow post-processing normalization of color profiles. Histogram matching and color correction algorithms were applied, with white balance and luminance adjusted according to the reference patches on the card. This procedure reduced the effects of lighting variability and camera sensor differences, ensuring greater consistency across the dataset.

#### Contrast enhancement using CLAHE

To improve the visibility of skin features, we applied Contrast Limited Adaptive Histogram Equalization (CLAHE) to all images. This method increases local contrast while avoiding excessive noise amplification, making it particularly suitable for medical images captured under controlled lighting. By enhancing subtle gradients of skin pigmentation, CLAHE helped make fine texture details more distinguishable for the learning algorithms.

#### Color space conversion

After contrast enhancement, images were transformed from the standard RGB space into alternative representations, specifically HSV (Hue, Saturation, Value) and YCbCr. These spaces provide a clearer separation between skin and non-skin regions, improving the reliability of skin detection. HSV is particularly effective for distinguishing colors based on hue and saturation, which facilitates isolating skin tones from surrounding areas. In contrast, YCbCr reduces the influence of illumination changes, offering a more consistent basis for detecting skin regions under varying lighting conditions. Together, these transformations enhanced the separation of skin from background elements and improved the accuracy of subsequent segmentation steps.

#### Skin detection and ROI extraction

To identify skin regions, a custom Python script (*skin_detection.py*) was applied to the transformed images. The script combined thresholding in both HSV and YCbCr spaces, followed by morphological operations to suppress non-skin areas. Nevertheless, because of variations in neonatal skin tones and minor inconsistencies in illumination, fully automated segmentation was not always sufficient for accurate region isolation. For this reason, trained operators manually refined the process by selecting ROIs from the segmented skin areas. Flat, evenly illuminated, and hairless regions of the face, abdomen, or inner forearm were prioritized. The selected ROIs were then cropped and resized to 224 × 224 pixels to match the input requirements of the deep learning models. This combined automated–manual approach ensured that the final images used for training and evaluation contained diagnostically relevant content.

#### Data augmentation techniques

To increase dataset diversity and mitigate overfitting, several augmentation techniques were applied during model training. These included: Random rotations within ± 15 degrees, Horizontal and vertical flips, Minor brightness and contrast shifts, Random zoom-in cropping. These transformations preserved class labels and biological plausibility while enhancing model generalizability to unseen cases. Data augmentation was implemented using the torchvision library and applied in real-time during training. After augmentation process, 792 high-quality images were retained for downstream analysis. Following augmentation, the dataset was balanced with an approximately equal number of jaundiced and non-jaundiced images in the training set. Augmentation was preferentially applied to the minority class to address class imbalance and improve model sensitivity. The dataset contained more non-jaundiced than jaundiced images. To reduce imbalance, we applied targeted augmentation to the jaundiced class (rotation, scaling, flipping, brightness/color jitter) and used class-balanced mini-batches during training. Augmentation was restricted to the training set; the test set reflected the natural prevalence and contained no augmented images.

**Fig. 6:**
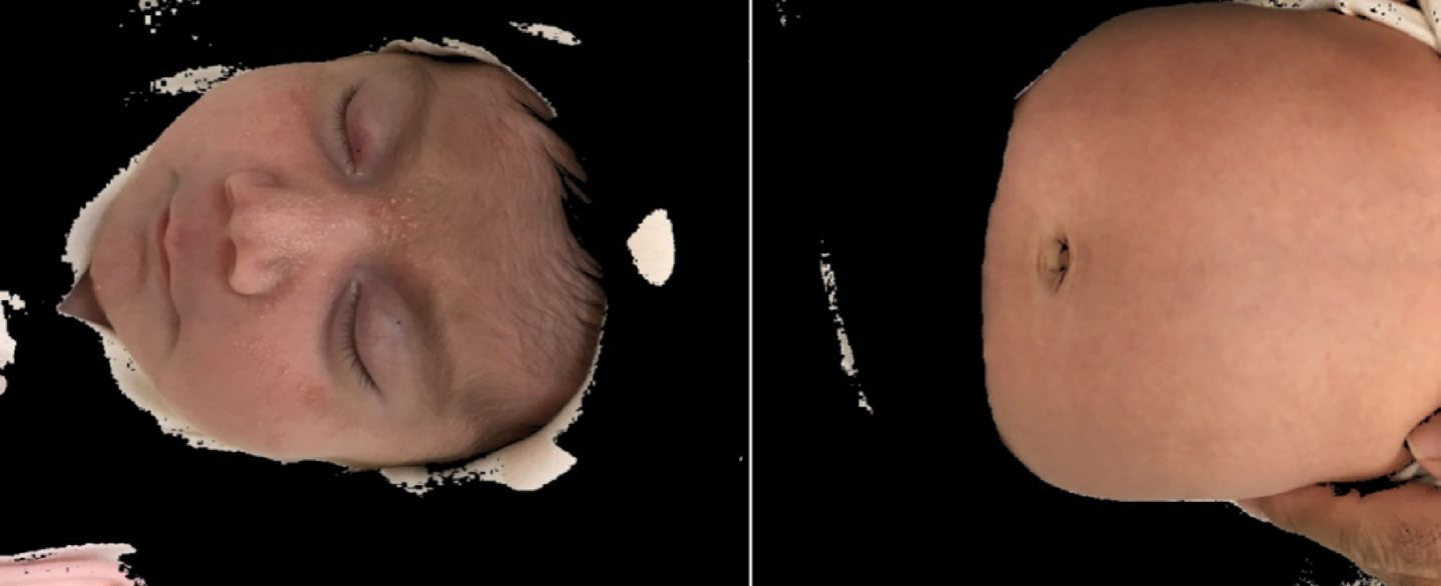
Output of the Skin Detection and ROI Selection process. In this image, non-skin areas have been successfully removed, leaving only the infant’s skin regions identified and separated. This step is used to enhance accuracy in diagnosis and analysis.

Data augmentation techniques (including rotation, flipping, and color jitter) were applied exclusively to the training set to increase variability and reduce overfitting. Augmentation was performed dynamically during training, ensuring that no augmented images were stored or carried over into the validation or test sets. This prevented any potential data leakage and guaranteed a fair performance evaluation. After applying the Skin Detection and ROI Selection techniques, Fig. 6 demonstrates the results of the process, where non-skin areas have been removed, isolating the skin regions for further analysis. These preprocessing steps not only aimed to highlight skin regions but also reduced noise, thus improving the overall clarity and quality of the images.

### Model architecture and training

**Fig. 7:**
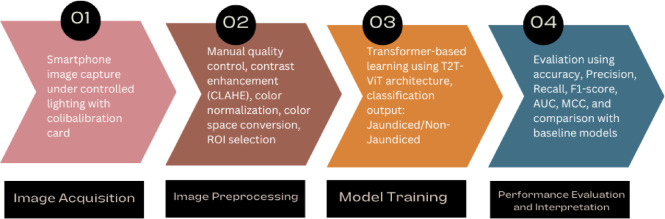
Overview of the neonatal jaundice detection pipeline, illustrating the sequential steps from image acquisition and preprocessing to model training and evaluation.

This section describes the architecture and training procedure of the proposed deep learning framework, as well as three baseline models employed for comparison: ResNet-50, SVM, and k-NN. All models were trained and tested on the same preprocessed neonatal skin images obtained from the defined ROIs, ensuring a consistent evaluation setting. The end-to-end workflow of the system, from image acquisition to classification, is summarized in Fig. 7.

#### Tokens-to-Token vision transformer (T2T-ViT)

**Fig. 8:**
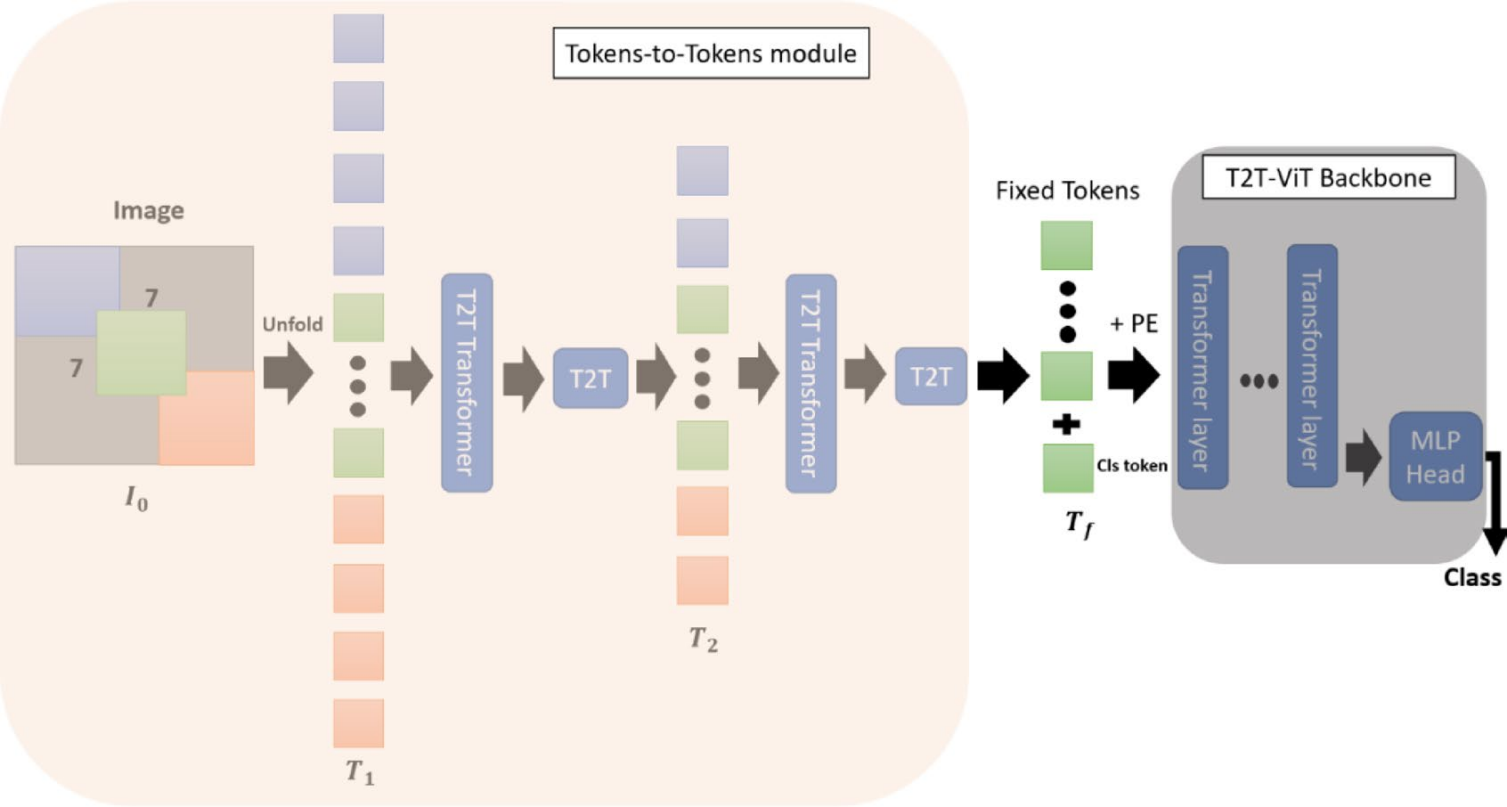
The overall architecture of T2T-ViT involves two main components. First, in the T2T-ViT module, the input image is initially divided into patches and then unfolded into a sequence of tokens, referred to as T0. This process progressively reduces the token length through multiple iterations, outputting Tf. These fixed-length tokens are then passed to the T2T-ViT backbone, which processes them to generate the final predictions. This structure efficiently transforms the input image data into a format suitable for prediction tasks.

The T2T-ViT is a recent development in transformer-based architectures designed for image classification. The structure, originally introduced by Yuan et al.^40^ and illustrated in Fig. 8, consists of two principal components: the T2T module, which converts the input image into tokens, and the T2T backbone, which processes these tokens through multiple transformer layers to generate the final prediction. Unlike the standard Vision Transformer (ViT), which partitions an image into fixed patches, the T2T module employs progressive soft splitting, preserving local detail and structural continuity. This property is particularly valuable in medical imaging, where subtle textures and fine spatial relationships are diagnostically important.

In this study, we implemented the T2T-ViT-14 configuration, which includes 14 transformer layers, a hidden dimension of 384, and an MLP size of 1536. Tokens were created using overlapping patch embedding with patch sizes of [7, 3, 3] and strides of [1, 1, 3]. The model was initialized with pretrained ImageNet weights and subsequently fine-tuned on our neonatal skin dataset to optimize performance for jaundice detection.

#### Loss function

For model optimization, we used the Binary Cross-Entropy with Logits Loss (BCEWithLogitsLoss), which is well-suited for binary classification tasks. This loss function combines a sigmoid activation with cross-entropy computation in a single step, providing numerical stability and efficient training. Its use enabled the T2T-ViT network to learn effective decision boundaries between jaundiced and non-jaundiced infants, thereby improving overall classification performance.

#### Training configuration

All deep learning experiments were implemented in PyTorch (v1.12.1) and executed on an NVIDIA RTX 4060 GPU with 16 GB of memory. The dataset comprised 792 neonatal skin images, of which 634 were allocated for training and 158 for testing. An 80/20 split was used to separate the data, without applying cross-validation in the current study. The dataset was divided into training (80%) and test (20%) sets. While this split provides an initial assessment of model performance, we recognize that a single random partition may introduce variability in the results. More robust evaluation strategies, such as k-fold or nested cross-validation, will be incorporated in future work to provide greater confidence in the reported metrics. While this approach provided a straightforward evaluation of performance, future work may incorporate k-fold cross-validation to achieve a more comprehensive assessment of model generalizability. Prior to training, all input images were resized to 224 × 224 pixels to match the expected input dimensions of the models. The model was trained for 300 epochs with a batch size of 8 and a learning rate of 0.0001, using the Adam optimizer and BCE With LogitsLoss. Although no early stopping mechanism was implemented in this study, validation performance was monitored throughout training to reduce the risk of overfitting. Future work will adopt early stopping or adaptive learning rate scheduling strategies to further optimize training duration and improve generalization. The Implementation summary for all models is detailed in Table 2.

**Table 2:** Training configuration and hyperparameters used for model development, including batch size, learning rate, number of epochs, optimizer type, and loss function.

Component	Description
Framework	PyTorch 1.12.1 (DL), Scikit-learn 1.1.1 (ML)
Hardware	NVIDIA RTX 4060 GPU, 16 GB
Image input size	224*224 pixels
Loss function	BCE With LogitsLoss
Optimizer	Adam
Learning rate	0.0001
Batch size	8
Epochs	300
Deep models	T2T-ViT-14, ResNet-50
ML models	SVM9RFB Kernel0, k-NN (k = 3)
Features (SVM/k**-**NN)	Statistical descriptors in HSV & YCbCr (mean, std, skew, kurtosis)
Augmentation	Rotation, flip, brightness shift, zoom-in cropping)
Classification type	Binary (Jaundiced/ Non-jaundiced)

### Baseline models

**Fig. 9:**
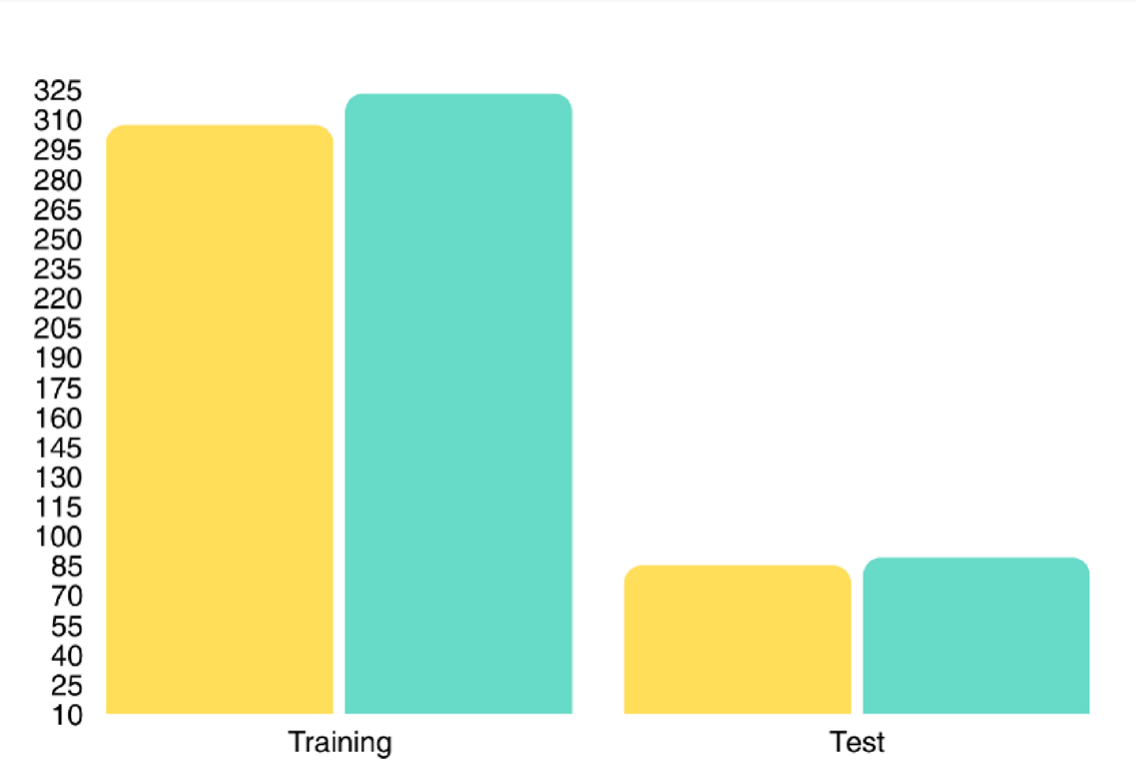
Class-wise distribution of jaundiced and non-jaundiced images in the training and test sets. The training set includes augmented samples, while the test set consists of original images only.

To benchmark the performance of the proposed transformer-based model, three widely used baseline approaches were implemented: a convolutional neural network (ResNet-50), a kernel-based classifier (Support Vector Machine, SVM), and a distance-based learner (k-Nearest Neighbors, k-NN). These models were chosen to represent different paradigms of machine and deep learning, providing a comprehensive basis for comparison. Figure 9 illustrates the class distribution of images in the training and test sets, reflecting the dataset composition after augmentation and ensuring a fair evaluation framework.

#### ResNet-50

ResNet-50, a widely adopted deep convolutional neural network, was chosen as a baseline because of its strong track record in medical image classification. The architecture consists of stacked residual blocks, which ease the training of deeper networks by enabling identity mappings and mitigating vanishing gradient problems. For this study, the network was adapted to a binary classification task by replacing the final fully connected layer with a single output node followed by a sigmoid activation. As with the T2T-ViT model, ResNet-50 was initialized with ImageNet-pretrained weights and subsequently fine-tuned on the neonatal skin image dataset under the same training configuration and hyperparameters.

#### Support vector machine (SVM)

SVM, originally introduced by Cortes^41^, is a well-established machine learning method applicable to both classification and regression. It is particularly effective in handling non-linear data distributions by applying kernel functions that project samples into higher-dimensional spaces, where linear separation becomes feasible^42^. In this study, an SVM with a radial basis function (RBF) kernel was implemented to model nonlinear relationships in the feature space. Feature vectors were derived from segmented skin ROIs and consisted of first-order statistical descriptors, including the mean and standard deviation of HSV and YCbCr channels, as well as skewness and kurtosis of the luminance (Y) component. All features were normalized using min–max scaling before classification. Model hyperparameters (C and γ) were optimized through grid search with five-fold cross-validation.

#### k-Nearest neighbor (k-NN)

The k-NN algorithm is a straightforward yet widely applied classification method that assigns labels based on the majority class among the nearest neighbors in the feature space^43^. In this study, the classifier employed Euclidean distance as the similarity measure. The value of *k* was systematically varied between 1 and 10, and the optimal setting was determined according to validation accuracy. As with the SVM model, classification was performed using color-based statistical features extracted from the ROIs. To reduce the influence of outlier neighbors, distance-weighted voting was incorporated^44^. All classical machine learning models, including k-NN, were implemented using the Scikit-learn library (v1.1.1) to ensure methodological consistency.

## Results

### Performance evaluation of deep Learning-Based models

Model performance was evaluated on the independent test set using a comprehensive suite of metrics, including accuracy, precision, recall (sensitivity), specificity, F1-score, Matthews correlation coefficient (MCC), and area under the curve (AUC). For the deep learning models that incorporated image feature extraction, additional indices such as root mean square error (RMSE) and peak signal-to-noise ratio (PSNR) were also assessed. All evaluations were performed on a hold-out set of 158 neonatal skin images, with a fixed decision threshold of 0.5. Confusion matrices were generated for each model to provide detailed insight into classification outcomes. Alongside point estimates, we report 95% confidence intervals (CIs) for accuracy, sensitivity, and specificity on the test set (*N* = 158; positives = 61, negatives = 97). Accuracy CIs were computed using the Wilson method; sensitivity and specificity CIs were computed using Clopper–Pearson intervals. For F1-score and MCC, exact CIs require case-level predictions and bootstrap resampling; since some baselines lack archived per-case outputs, these metrics are reported as point estimates and clearly marked as such. We plan to provide bootstrap CIs for F1 and MCC in future work once per-case predictions are available. Receiver operating characteristic (ROC) curves were plotted, and AUC values were computed using the trapezoidal method. Table 3 summarizes the classification performance of ResNet-50 and the proposed T2T-ViT model on the test set:

**Table 3:** Comparison of ResNet-50 and T2T-ViT based on evaluation metrics.

	Accuracy	Precision	Specificity	Recall	F1Score	MCC	AUC	RMSE	PNSR (dB)
T2T-ViT	0.99	0.99	0.99	0.99	0.99	0.98	0.99	-	-
REsNet	0.85	0.90	0.92	0.78	0.83	0.70	0.85	0.38	8.18

Across nearly all metrics, the proposed T2T-ViT model outperformed the ResNet baseline. ResNet achieved relatively high specificity (0.92) and precision (0.90), but its recall (0.78) indicated a higher risk of false negatives. In contrast, the T2T-ViT attained more balanced performance, as reflected in its superior F1-score and MCC values. The difference was most striking in discriminative capacity: while ResNet achieved an AUC of 0.85, the T2T-ViT reached 0.99, underscoring its robustness and reliability for neonatal jaundice detection. RMSE and PSNR were reported for ResNet to capture reconstruction fidelity, but these metrics were not applicable to the T2T-ViT since its architecture performs direct classification without intermediate image generation.

**Fig. 10:**
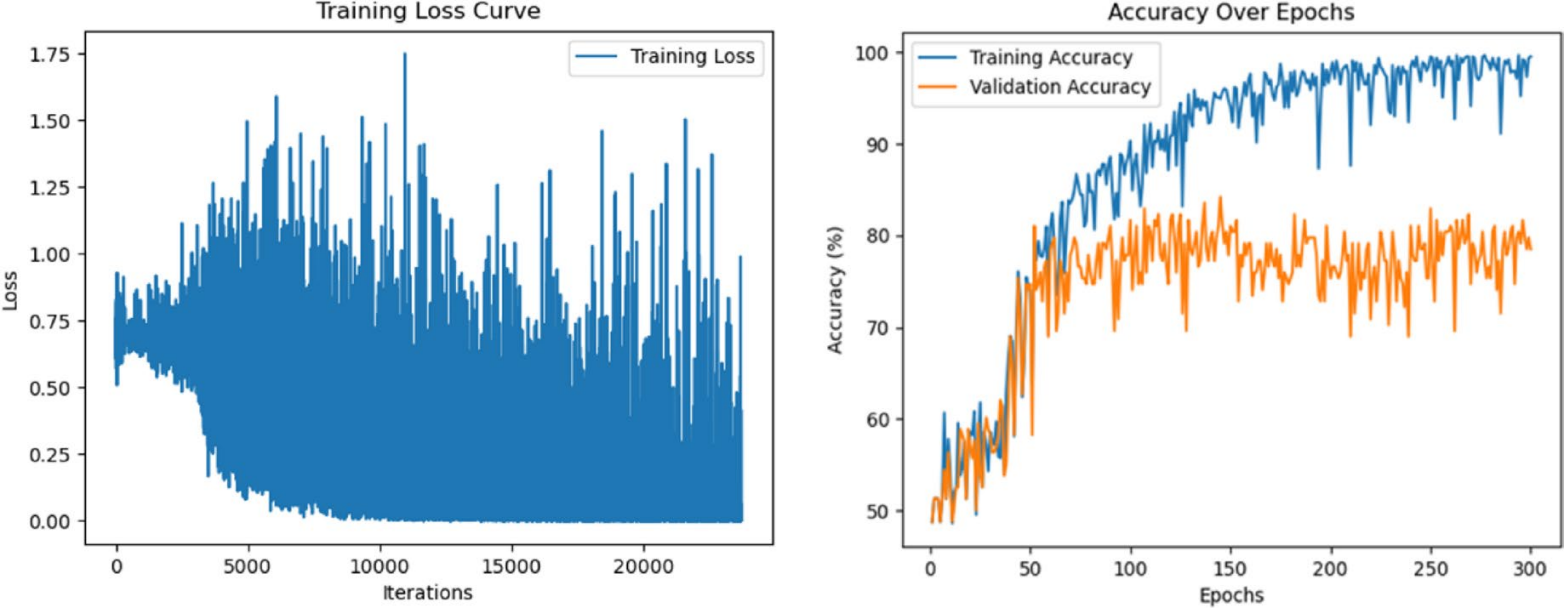
Training loss curve of the T2T-ViT model over 300 epochs, during the model’s learning process (left) and training/validation accuracy curve over 300 epochs (right).

Figure 10 illustrates the learning curves of the T2T-ViT model across 300 epochs. The training accuracy increased steadily and approached nearly 100%, indicating strong fit to the training set. In contrast, validation accuracy fluctuated considerably and stabilized around 80% after approximately 150 epochs. The widening gap between the two curves in later epochs suggests a degree of overfitting, with the model capturing training patterns more effectively than generalizable features. Although data augmentation and manual ROI selection helped to reduce overfitting, the irregular validation trajectory implies that further regularization strategies, such as dropout, early stopping, or weight decay, may be needed to improve generalization.

**Fig. 11:**
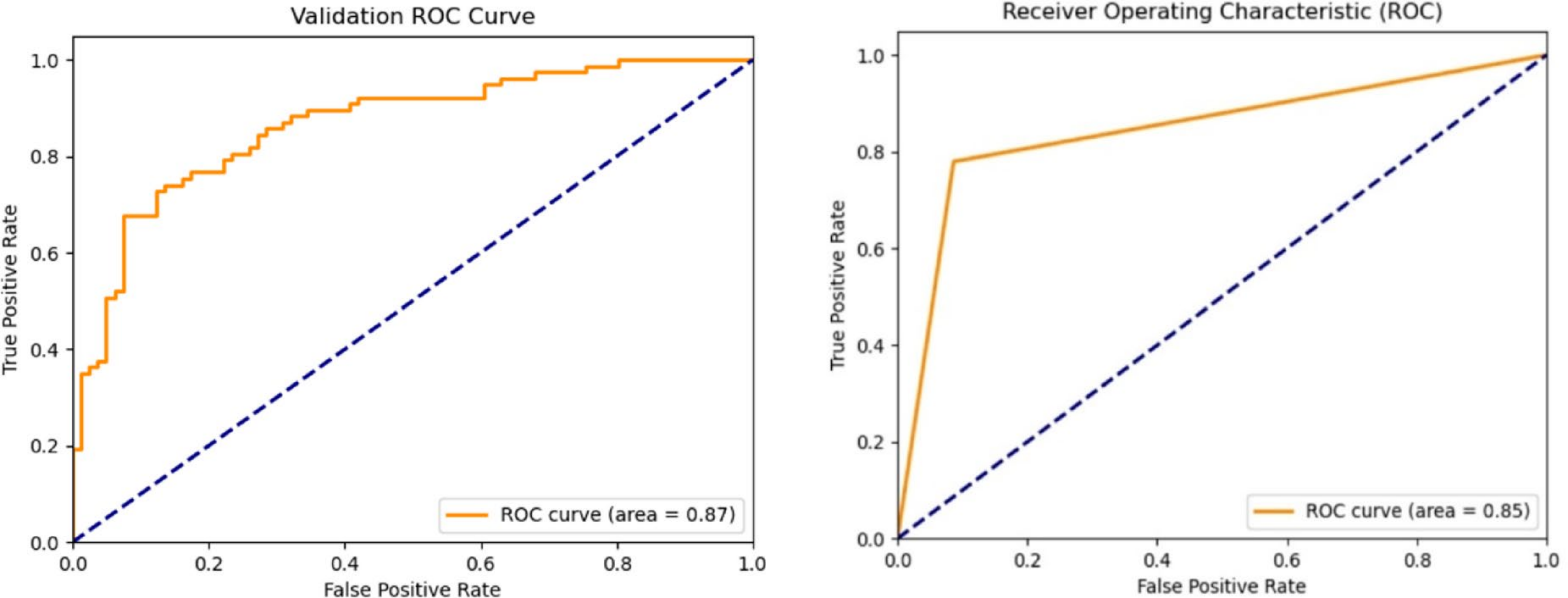
The ROC curve of T2T-ViT model (left), and ROC curve of ResNet model (right) for the validation set.

Figure 11 shows the ROC curves of the T2T-ViT and ResNet models on the validation set. The T2T-ViT achieved an AUC of 0.99, indicating excellent discriminative ability and a strong balance between sensitivity and specificity. In contrast, ResNet obtained an AUC of 0.85, reflecting weaker separation between jaundiced and non-jaundiced cases. The high and smooth ROC profile of T2T-ViT further supports its robustness in distinguishing clinical cases with minimal false positives and false negatives.

### Confusion matrices and metric analysis

**Fig. 12:**
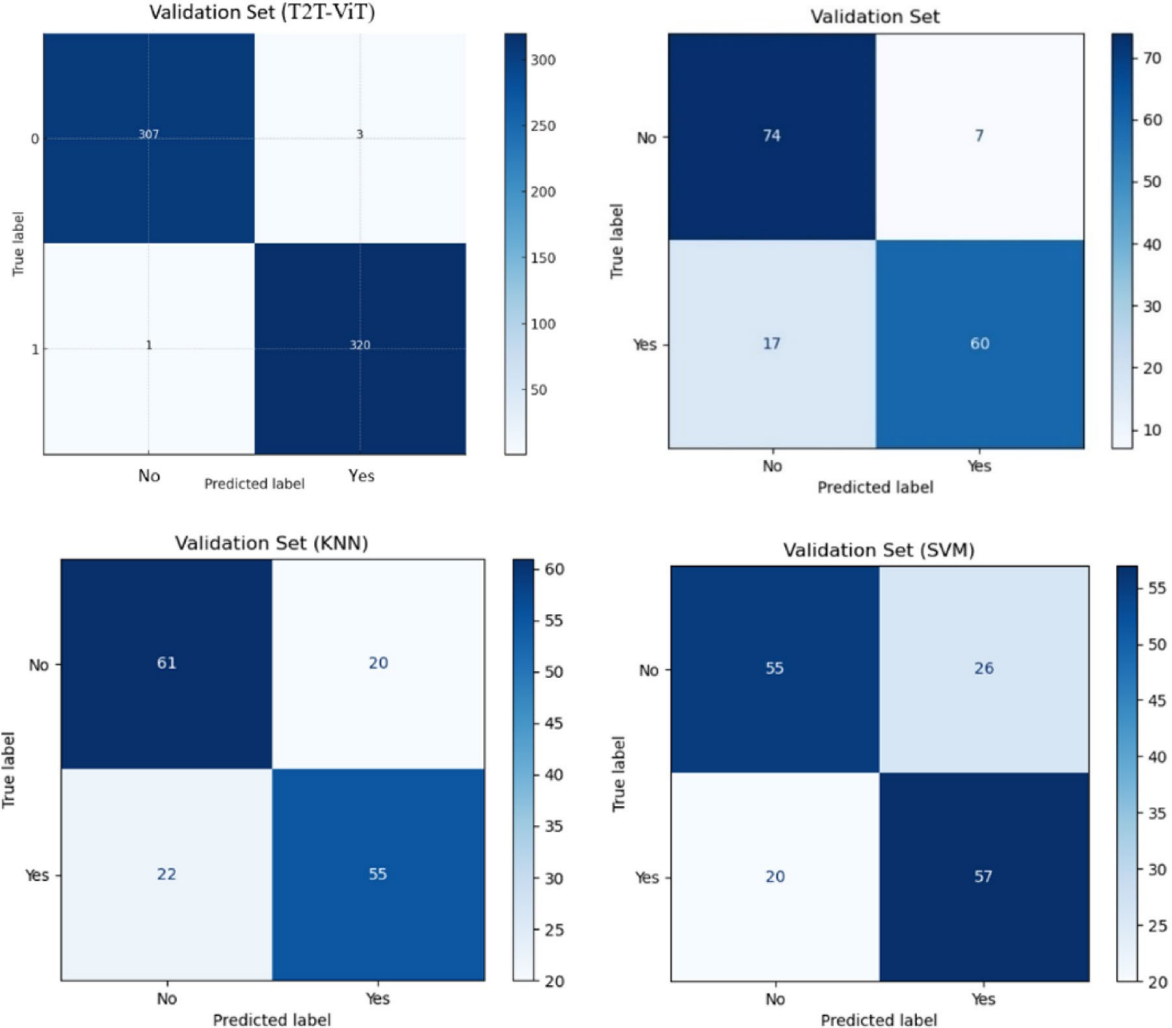
Compares the performance of k-NN, SVM, ResNet, and T2T-ViT models using confusion matrices on the validation set.

Figure 12 presents the confusion matrices of the four evaluated models (k-NN, SVM, ResNet-50, and T2T-ViT) on the validation set. Both k-NN and SVM exhibited higher rates of misclassification, particularly for jaundiced cases, reflecting their limited ability to capture subtle chromatic differences. ResNet-50 achieved good overall accuracy and strong specificity but showed reduced sensitivity, leading to more false negatives. In contrast, T2T-ViT correctly classified nearly all cases (320 jaundiced and 307 non-jaundiced), with only four misclassifications in total. These results highlight the superiority of the transformer-based approach in balancing sensitivity and specificity, minimizing missed jaundiced cases compared to the classical and CNN-based baselines.

### Results of classical methods

**Fig. 13:**
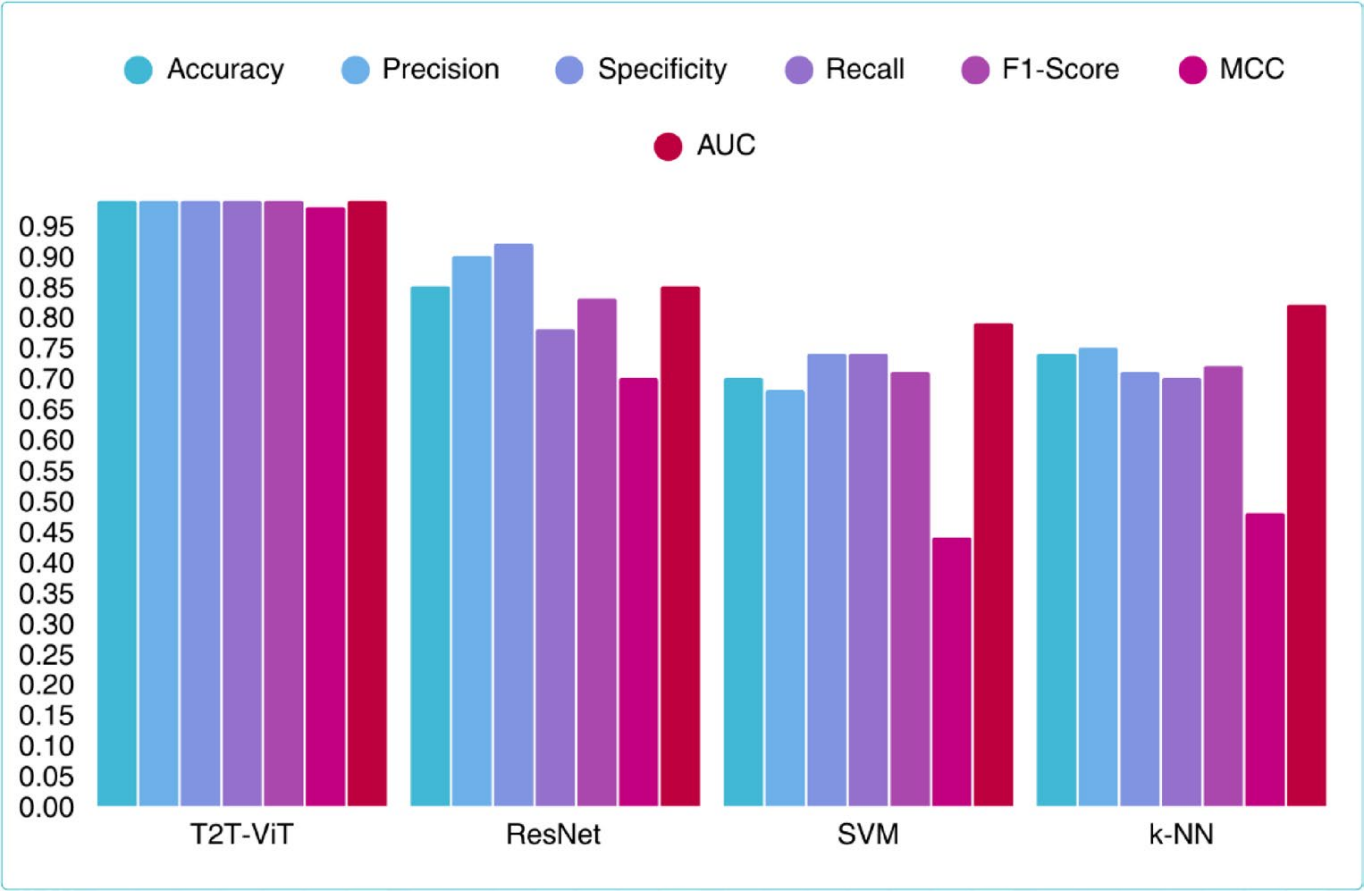
Comparison of classification performance across four models (T2T-ViT, ResNet-50, SVM, and k-NN) based on key evaluation metrics including accuracy, precision, specificity, recall, F1-score, MCC, AUC.

To contextualize the performance of the proposed T2T-ViT model, we compared it against classical machine learning approaches (SVM and k-NN) and the ResNet-50 baseline. All models were trained and evaluated on the same preprocessed dataset using identical performance metrics. Table 4 summarizes the results, highlighting the consistently superior performance of T2T-ViT across accuracy, recall, F1-score, and MCC. To complement these results, Table 5 reports 95% confidence intervals (CIs) for accuracy, sensitivity, and specificity across all models. These intervals quantify uncertainty and provide additional robustness assessment. For instance, the proposed T2T-ViT achieves Accuracy = 0.994 (95% CI: 0.965–0.999), Sensitivity = 0.984 (0.912–1.000), and Specificity = 0.990 (0.944–1.000), reflecting high stability compared to traditional baselines. Figure 13 provides a comparative visualization of the main evaluation metrics for all four models, clearly emphasizing the effective performance of the T2T-ViT model across all metrics. For clarity, a consolidated summary table has been included as Table 6. This table presents accuracy, recall (all with 95% CIs), and F1-score and MCC (point values) for all evaluated models. This comparative table highlights the superior performance of the proposed T2T-ViT relative to both classical machine learning methods and CNN-based approaches.

**Table 4:** Comparative performance of T2T-ViT, ResNet-50, SVM, and k-NN in main metrics.

	Accuracy	Precision	Specificity	Recall	F1Score	MCC	AUC
T2T-ViT	0.99	0.99	0.99	0.99	0.99	0.98	0.99
REsNet	0.85	0.90	0.92	0.78	0.83	0.70	0.85
SVM	0.70	0.68	0.74	0.74	0.71	0.44	0.79
K-NN	0.74	0.75	0.71	0.70	0.72	0.48	0.82

**Table 5:** Robustness assessment of all models with 95% confidence intervals (CIs) for accuracy, sensitivity, and specificity on the test set (*N* = 158; 61 jaundiced, 97 non-jaundiced).

Model	Accuracy (95% CI)	Recall/Sensitivity (95% CI)	Specificity (95% CI)
T2T-ViT	0.994 (0.965–0.999)	0.984 (0.912–1.000)	0.990 (0.944–1.000)
ResNet-50	0.854 (0.791–0.901)	0.787 (0.663–0.881)	0.918 (0.844–0.964)
SVM	0.703 (0.627–0.768)	0.738 (0.609–0.842)	0.742 (0.643–0.826)
k-NN	0.741 (0.667–0.803)	0.705 (0.574–0.815)	0.711 (0.610–0.799)

**Table 6:** Consolidated summary of classification performance (quick reference). Accuracy and recall are reported with 95% confidence intervals; F1-score and MCC are reported as point estimates.

Model	Accuracy (95% CI)	Recall (95% CI)	F1-score	MCC
T2T-ViT	0.994 (0.965–0.999)	0.984 (0.912–1.000)	0.99	0.98
ResNet-50	0.854 (0.791–0.901)	0.787 (0.663–0.881)	0.83	0.70
SVM	0.703 (0.627–0.768)	0.738 (0.609–0.842)	0.71	0.44
k-NN	0.741 (0.667–0.803)	0.705 (0.574–0.815)	0.72	0.48

## Discussion

### Interpretation of results

The comparative evaluation of the four models, T2T-ViT, ResNet-50, SVM, and k-NN, highlights clear differences in their ability to detect neonatal jaundice from skin images. These differences arise from the models’ architectural designs and the extent to which they can extract informative features from subtle color variations. While classical approaches such as SVM and k-NN depend on handcrafted descriptors and simple decision boundaries, deep learning models provide stronger feature learning capacity.

Among all tested methods, the T2T-ViT achieved the highest and most balanced performance, with accuracy, precision, recall, F1-score, and AUC all close to 99%, and an MCC of 0.98. This consistency indicates that the model can reliably capture fine chromatic gradients and local textural cues, which are essential for identifying mild or early-stage jaundice. By contrast, ResNet-50, despite its depth and residual connections, achieved lower recall (0.78) and MCC (0.70), reflecting a greater risk of misclassifying jaundiced infants as healthy, a clinically critical error.

Beyond achieving superior numerical performance, the proposed T2T-ViT model also offers clinical and practical advantages. Its attention-driven architecture enhances sensitivity to subtle chromatic cues, enabling early detection of mild jaundice. The hierarchical token-to-token mechanism improves stability under variable imaging conditions, while the lightweight design and smartphone compatibility make it a cost-effective and portable tool for large-scale screening, particularly in resource-limited settings. However, as the study was conducted under controlled conditions with a single device, factors such as lighting variability, device quality, and healthcare infrastructure must be addressed. Future work will therefore focus on multi-center validation and cost-effectiveness analysis to ensure scalability and integration into real-world clinical workflows. The classical models performed notably worse: SVM reached 70% accuracy with limited precision and recall, while k-NN achieved slightly better results (74% accuracy, MCC 0.48) but still struggled with generalization. Their confusion matrices confirmed higher rates of false positives and false negatives, underscoring their limited suitability for clinical deployment. Taken together, these findings show that transformer-based architectures, and in particular T2T-ViT, provide a more reliable and comprehensive solution for jaundice detection compared to both CNN-based and traditional machine learning methods.

### Comparative analysis with recent deep learning approaches

Recent studies have explored deep learning techniques for non-invasive neonatal jaundice detection using skin images. A structured comparison of these approaches with our work is presented in Table 7, which contrasts differences in modality, architecture, cohort, validation strategy, and clinical applicability. For example, methods such as SSGNN^20^and SPAGNN/SPEGNN^45^reported high accuracies of 93% and 96.5%, but provided limited information on dataset size or imaging standardization. A model based on ImageNet features achieved 90.56% accuracy but only 67.8% sensitivity^46^, underscoring the clinical risk of missing true positive cases. Abdulrahman et al.^47^applied a ResNet-50 model, obtaining an accuracy of 84.09%, while another study^48^reported unrealistically low performance (1.22%) on a very small dataset of 20 samples, highlighting challenges of generalization. In contrast, our T2T-ViT achieved substantially higher and more consistent performance across all key metrics (accuracy, sensitivity, precision, F1-score, and AUC), using a clinically curated dataset of 792 images collected under standardized conditions. This suggests a more reliable foundation for practical applications.

**Table 7:** Comparison of the main characteristics of studies that used the deep learning method.

Reference	country	Body region	Model/Method	Cohort / Target Group	Dataset Size	results	Key Limitations	Distinction from Our Work
^20^	India	Face	SSGNN	Neonates	Not reported	Accuracy: 93.00	Reliance on traditional regression; limited accuracy; no robust statistical evaluation	We employ a Vision Transformer; more comprehensive metrics and better results
^35^	India	facial regions (face, forehead) or sternum	CNN + Color Card	Neonates	Not clearly reported	Acc. ~93%	Requires external calibration; small dataset	Our work does not need calibration tools; uses Transformer attention end-to-end
^36^	Iran	skin regions (head, face, chest)	1D CNN	Neonates	Moderate (exact N not stated)	Reported good prediction accuracy	Non-image based; not smartphone-ready	Our approach is fully image-based and directly smartphone-compatible
^38^	Saudi Arabia	Eye, face	Transfer Learning (VGG-16) + ML (MLP, SVM, RF, DT)	Neonates	100	skin: Acc. 86.8%, Eye: 79%, Fusion: ~80%	Very small dataset; strong dependence on calibration card	Our work applies Transformer on larger neonatal dataset without feature fusion
^45^	India	Not specified	SPAGNN, SPEGNN	Neonates	Not reported	Acc.: 96.5%, Sens.: 96.6%, Prec.: 95%, AUC: 97.2	Relies on facial and scleral images, which may be more variable and harder to capture; graph Neural Networks (GNNs) are relatively complex but may not outperform ViT in capturing global skin tone patterns; limited clinical validation, mostly algorithmic focus.	We employed Vision Transformer (T2T-ViT), which is specifically strong for image-based tasks compared to GNNs
^46^	Taiwan	sternum	ImageNet-based DL	Neonates	228	Acc.: 90.56%, Sens.: 67.8%, Spec.: 96.9%, Prec.: 85.08%,	Relied on a dynamic white balance threshold rather than advanced deep learning; limited validation, no transformer-based or large-scale comparative analysis; performance sensitive to device setting and illumination	We applied a Vision Transformer; large dataset and more comprehensive evaluation
^47^	Iraq	face	ResNet-50	Neonates	145	Accuracy: 84.09	Limited accuracy; relies on a basic CNN model; dataset size and diversity not clearly described; no use of color calibration card or standardized imaging; no external test set mentioned	We reach better results with a Transformer model; dataset included both healthy and jaundiced neonates, collected under controlled protocol
^48^	Australia	face	Deep learning technique	Neonates	20	Accuracy: 91.22%	Dataset size relatively small; limited validation and real-world clinical testing	Applied color calibration card to ensure standardized color representation; larger dataset (500 neonates, controlled imaging); evaluation with multiple ML/DL baselines (CNN, SVM, k-NN) for fairness.

Beyond outperforming recent CNN-based approaches, our model also surpassed traditional machine learning methods such as SVM and k-NN^49^, which are prone to higher misclassification rates and limited capacity to capture complex image features. These results emphasize the advantages of transformer-based architectures when combined with carefully controlled data acquisition. Importantly, the T2T-ViT achieved not only higher overall accuracy but also a better balance between sensitivity and specificity, reducing the risk of misclassification in borderline cases. The method’s reliance on standard smartphone cameras further supports its potential for cost-effective use in low-resource clinical settings or at-home monitoring.

Unlike prior CNN-based studies that rely heavily on local feature extraction or handcrafted features, our transformer-based approach leverages token-to-token attention to capture both local and global dependencies, leading to improved robustness under variable conditions. Moreover, the standardized imaging setup and inclusion of neonates with varying skin tones help address several limitations reported in earlier studies. Taken together, this work provides evidence that transformer-based pipelines can serve as a practical and scalable solution for early jaundice screening in clinical and community contexts.

### Limitations and future directions

This study has several limitations that should be acknowledged. First, all images were collected at a single clinical center using one device (iPhone 6 S) under controlled lighting. While this ensured consistency, it limits generalizability to other devices and real-world environments. Differences in smartphone hardware, including resolution, sensor quality, and color calibration, may alter image properties and affect performance. Future studies should therefore include multi-center cohorts and a wider range of devices (e.g., Android models and lower-quality cameras), and explore device-independent calibration strategies to support robust cross-device applicability.

Second, the dataset was restricted to Iranian neonates, representing a relatively narrow range of skin pigmentation. This raises concerns about generalizability to more diverse populations. Expanding data collection across multiple centers and ethnic groups will be important to ensure fairness and broader applicability. Similarly, preterm infants were not included, despite differences in skin maturity that may influence image characteristics. Addressing these gaps is necessary for the model to be clinically comprehensive.

Third, the ground truth relied on a single serum bilirubin measurement at the time of imaging, without follow-up testing for borderline or fluctuating cases. A uniform bilirubin threshold of 15 mg/dL was also applied, which, while simplifying this proof-of-concept study, does not reflect guideline-based thresholds tailored to gestational age and postnatal age. Although this fixed cutoff was used for methodological consistency, all diagnostic classifications were reviewed and confirmed by a pediatric specialist, ensuring clinical accuracy in labeling. Future work will incorporate guideline-specific cutoffs (e.g., AAP or NICE nomograms) and repeated measurements to improve diagnostic accuracy.

From a methodological perspective, some constraints remain. Although 95% confidence intervals were reported for accuracy, sensitivity, and specificity, they could not be computed for F1-score and MCC due to data aggregation. Future work will apply bootstrap resampling to estimate these intervals. The dataset also contained fewer than two images per subject on average (792 images from 500 neonates), which restricts the training capacity of transformer-based models and increases overfitting risk. While data augmentation and regularization helped, external validation is still needed. In addition, ROI extraction was performed manually after automated preprocessing, introducing operator dependence and limiting scalability. Developing a fully automated end-to-end pipeline remains an important next step.

The training protocol also had limitations. A fixed 300-epoch schedule without early stopping was used, which may not represent the most efficient or robust approach. Future work will explore adaptive strategies such as early stopping, dynamic learning rate scheduling, or weight decay to improve training efficiency. Furthermore, evaluation relied on a single 80/20 train–test split. More robust methods such as k-fold or nested cross-validation will be adopted in future studies to reduce sensitivity to data partitioning and provide more reliable performance estimates.

Looking ahead, several directions can extend the clinical utility of this work. Future models could predict jaundice severity rather than a binary label, supporting more informed clinical decision-making. Incorporating additional clinical features (e.g., birth weight, gestational age, hemoglobin) could enable multi-modal systems that integrate physiological and visual cues. Explainable AI methods, such as attention map visualization, may also enhance interpretability and clinician trust. Finally, deployment in a mobile application with an embedded preprocessing pipeline could enable real-time, at-home screening, particularly in low-resource settings, reducing the burden on healthcare systems.

## Conclusion

This study introduced a practical and non-invasive diagnostic system for neonatal jaundice detection based on the T2T-ViT transformer architecture. Leveraging images acquired under controlled but practical conditions, the system demonstrated feasibility without the need for specialized hardware. The proposed model achieved high performance across multiple metrics, outperforming both classical machine learning approaches and the ResNet-50 baseline. These findings highlight the potential of transformer-based models in capturing subtle chromatic features critical for jaundice detection. While some manual preprocessing was still required, the pipeline remains simple and adaptable to further automation. With broader validation and integration of explainable AI, this framework has the potential to evolve into a cost-effective and accessible tool for early neonatal jaundice screening, particularly in low-resource clinical settings.

## Data Availability

The dataset generated and/or analyzed during the current study are not publicly available due to patient privacy concerns and institutional ethical regulations. However, de-identified datasets and additional materials can be made available from the corresponding author upon reasonable request and subject to ethical approval.
